# A Clinical, Radiographic and Histological Study of Unerupted Teeth in Dogs and Cats: 73 Cases (2001–2018)

**DOI:** 10.3389/fvets.2019.00357

**Published:** 2019-11-08

**Authors:** Emma Bellei, Silvia Ferro, Eric Zini, Margherita Gracis

**Affiliations:** ^1^Department of Veterinary Medical Science, Alma Mater Studiorum-Bologna University, Bologna, Italy; ^2^“I Portoni Rossi” Veterinary Hospital, Bologna, Italy; ^3^Department of Comparative Biomedicine and Food Science, University of Padova, Padova, Italy; ^4^Clinic for Small Animal Internal Medicine, Vetsuisse Faculty, University of Zurich, Zurich, Switzerland; ^5^Department of Animal Medicine, Production and Health, University of Padova, Padova, Italy; ^6^Department of Internal Medicine, Istituto Veterinario di Novara, Novara, Italy; ^7^Department of Dentistry and Oral Surgery, Istituto Veterinario di Novara, Novara, Italy; ^8^Department of Dentistry and Oral Surgery, San Siro Veterinary Clinic, Milan, Italy

**Keywords:** unerupted teeth, dentigerous cyst, neoplasia, eruption, dog, cat

## Abstract

Lack of dental eruption may be accompanied by development of dentigerous cysts and has also been rarely associated with neoplasia. However, little information is available on prevalence of unerupted teeth and associated lesions in dogs and cats. The main objective of this study was to describe the epidemiologic data of canine and feline dental patients with unerupted teeth, and assess the prevalence of associated dentigerous cysts and tumors. Secondary aims included the evaluation of possible factors implicated in cystic development, and description of the histological features of dentigerous cysts. Medical and dental records, intraoral photographs, intraoral radiographs of client-owned dogs and cats with clinically missing teeth examined between 2001 and March 2018 were reviewed. Collected data included signalment, reason for presentation, number, type, depth of inclusion and angulation of unerupted teeth, presence of cystic lesions or tumors, abnormalities affecting involved teeth, histopathological findings, performed treatment and outcome. Seventy-three animals (69 dogs and 4 cats) with 113 unerupted teeth were included. The most frequent unerupted tooth in dogs was the first premolar teeth (78%), followed by the canine and third molar teeth. Dentigerous cysts were diagnosed associated with 48 (44.4%) teeth in dogs and one out of five unerupted teeth in cats. The affected teeth in dogs were predominantly in horizontal inclination (40%) and in soft tissue inclusion (77%). Brachycephalic canine breeds were overrepresented. The only unerupted tooth in boxer dogs was the first premolar tooth (32 teeth). Ninety percentage of boxers with unerupted teeth developed associated lesions (25 dentigerous cysts and one tumor). Two ameloblastomas (one in a dog and one in a cat) and one osteosarcoma (in a dog) were diagnosed in association with three unerupted teeth. Histology was essential in diagnosing two odontogenic cysts not evident on radiographs. In all cases that were followed-up, treatment (i.e., extraction, extraction and surgical curettage, or operculectomy) appeared successful. Untreated dentigerous cysts showed progression at re-examination. None of the unerupted teeth without evidence of cyst at the time of diagnosis showed incipient cystic development. None of the evaluated factors were associated with lack of eruption and/or development of associated lesions.

## Introduction

An unerupted tooth is a tooth that does not emerge into the mouth within the expected time frame because of lack of space, malposition, and other physical impediments such as the presence of a tumor or fibrous tissue along the path of eruption (impacted tooth), or because of a lack of eruptive forces (embedded tooth) (1).

Clinical, radiographic and pathological features associated with unerupted teeth are well-described in humans (2), but only a few reports are present in the veterinary literature.

In human patients, the prevalence varies considerably in different countries (from 8 to 38%) (3, 4). The most frequently affected teeth are the mandibular and maxillary third molar (up to 68.6% of the cases), followed by the maxillary canine, mandibular and maxillary premolar teeth, and maxillary central incisor teeth (5). In humans, 1.4% of unerupted teeth develop dentigerous cysts (3) and <1% may be associated with tumor development (6). Unerupted teeth are typically asymptomatic, but pain may be reported because of inflammation of the soft tissues surrounding the crown (pericoronitis), or pressure resorption of adjacent roots (1). Diagnostic imaging (i.e., radiography or computed tomography) is required to confirm the presence of an unerupted tooth in edentulous areas (3). Extraction is the treatment of choice in case of loss of bone around the impacted teeth, presence of cysts or tumors, displacement of the adjacent teeth, or chronic pain (1). Sometimes, treatment is recommended even in the absence of clinical or radiographic pathologic findings, as a preventive method against the development of cystic lesions that can lead to severe loss of surrounding bone and resorption of adjacent teeth (7). However, because of the proximity of many unerupted third molar teeth to the inferior alveolar nerve and the risk of nerve injury during extraction, in humans coronectomy and intentional root retention of healthy, unerupted teeth has also been recommended (8). Furthermore, surgical exposure and orthodontic treatment may be attempted in select cases (5).

Little information is available on prevalence of unerupted teeth in companion animals. Only a few cases have been described in cats (9–12). In dogs, most of the studies and case reports have focused on the description of associated dentigerous cysts, with a reported prevalence of 29.1% (13, 14). Brachycephalic breeds (e.g., boxer, Boston terrier, pugs, and Shi-tzu) have been described as more frequently affected, but a variety of breeds of different morphology (e.g., Belgian malinois, German shepherd, Labrador retriever, Dalmatian dog, and greyhound) have also been reported. Based on the available information, the permanent first premolar tooth seems to be the most affected tooth in dogs, followed by the canine teeth (13, 14). Several criteria, including the presence of a coronal radiolucency extending apically beyond the cementoenamel junction, cortication, bone expansion, and root or bone resorption of adjacent teeth, have been suggested for the radiographic identification of cystic lesions (14). However, in absence of more sensitive diagnostic imaging systems such as cone beam computed tomography (CBCT) or standard CT, the radiographic diagnosis of cystic development may be difficult to obtain in case of small lesions. A method comparing the size of the periodontal ligament space of a reference tooth and the pericoronal lucency of unerupted teeth has been suggested to differentiate between small dentigerous cysts and normal dental follicles (14). Nevertheless, the applicability of this criterion remains to be confirmed.

Extraction of embedded and impacted teeth is recommended in veterinary patients when related pathology (i.e., dentigerous cyst, bone, and root resorption) is diagnosed (14). However, indications for the correct approach to unerupted teeth in the absence of clinical and radiographic signs of complications are lacking in the veterinary literature.

The main purpose of this retrospective study was to describe canine and feline dental patients with unerupted teeth and assess the prevalence of associated dentigerous cysts and tumors. As secondary aim, possible factors implicated in cystic development were evaluated. Furthermore, the radiographic and histological features were defined and compared in 10 cases to evaluate if the size of the pericoronal radiolucency in radiographically doubtful cases could help reaching a definitive diagnosis of cystic development.

## Materials and Methods

Medical and dental records, intraoral photographs and radiographs of client-owned dogs and cats examined between February 2001 and March 2018 at different veterinary clinics in northern Italy were reviewed. All patients were presented because of an oral or maxillofacial problem, but not necessarily because of missing teeth. Complete examination of the head and oral cavity was performed under general anesthesia. Patients were included in the study if full mouth intraoral radiographic examination or at least radiographic examination of all edentulous areas had been performed, and if at least one unerupted tooth was radiographically present.

Data collected for each patient included: signalment (species, breed, sex, age, and body weight at the time of diagnosis), main reason for presentation (i.e., related to missing tooth/teeth or not), number and type of unerupted teeth, presence or absence of clinically and/or radiographically visible signs indicative of cystic lesions (i.e., a well-defined radiolucency around or beside the crown of an unerupted tooth), presence of neoplastic lesions associated with unerupted teeth, histopathological findings (for biopsied lesions), depth of inclusion (i.e., gingival vs. bone inclusion) and inclination of the unerupted teeth in the jaw, radiographic abnormalities affecting unerupted tooth/teeth and adjacent teeth, treatment performed, treatment outcome, and follow-up findings.

**Figure 1:**
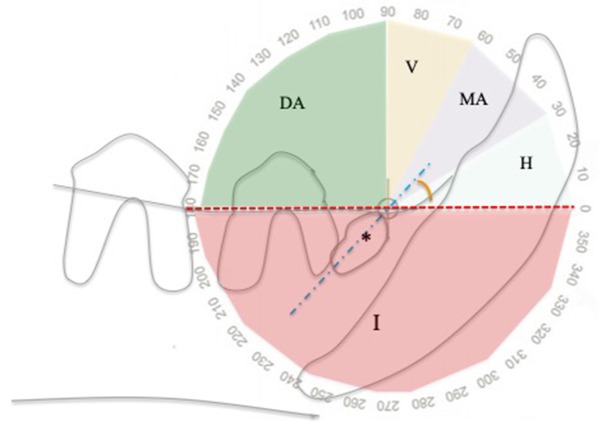
Angle of inclination of unerupted teeth relatively to the alveolar margin, using a modified Winter classification (15). This example shows an unerupted right mandibular first premolar tooth (asterisk) in mesioangular inclination. DA, distoangular; H, horizontal; I, inverted; MA, mesioangular; V, vertical; Blue dotted line, unerupted tooth long axis; Red line, alveolar margin.

To describe the inclination of the unerupted tooth within the jaw, the classification system by Winter was utilized in this study, typically used to score the difficulty of impacted molar extraction in humans (15). The inclination, measured as the angle between the long axis of the tooth and the occlusal plane, may be horizontal (0°-30° in mesial direction), mesioangular (31°-60° in mesial direction), vertical (61°-90° in mesial direction), distoangular (91°-180° in distal direction), inverted (negative angle <0°, with the crown pointing in the opposite direction to the occlusal plane) (15) (Figure 1).

Teeth were considered in soft tissue inclusion when one side (normally the side toward the alveolar margin) was covered only by gingival tissue, and in bone inclusion when they were completely encased in bone (tooth not visible after cutting the overlying gingiva and/or radiographically surrounded by bone) (16).

Treatments performed included: operculectomy (for teeth in gingival inclusion), extraction of unerupted teeth only (if cystic lesions were not evident clinically and/or radiographically), extraction of unerupted teeth and curettage of the cystic lining, and maxillectomy/mandibulectomy including the unerupted teeth and cystic lesion (in case of concomitant presence of neoplasia). In some cases, treatment was not performed, but radiographic monitoring was recommended.

For histological evaluation, samples were fixed in 10% buffered formalin and paraffin embedded. Then, 4 μ sections were stained with hematoxylin and eosin, and observed with an optical microscope (Olympus BX40, Segrate, Milan, Italy). Mineralized tissues were decalcified after fixation with a rapid decalcifying solution (Biodec R, Bio-opotica, Milan, Italy). A single pathologist (SF) examined all histological samples.

Outcome of treatment was evaluated clinically and whenever possible radiographically at the last follow-up available. The possible outcomes were: complete healing, absence of healing, or recurrence. The lesion was classified as completely healed if, at the time of the last follow-up examination available, there was absence of swelling or any other type of soft tissue abnormality [except for the presence of mild inflammation and suture material along the surgical wound, which was considered normal in case of short-term (<4 weeks) follow-ups] and, when available, the radiographic examination showed evidence of new bone formation and absence of any radiolucent lesions indicative of a cystic process at the previous surgical site.

Absence of healing at the time of the last follow-up examination available was determined by clinical evidence of flap's dehiscence, presence of swelling, persistent (>4 weeks) soft tissue inflammation or any other type of soft tissue abnormality at the surgical site, and/or radiographic persistence of a radiolucent lesion or a radiopaque, tooth-like material following treatment.

If, following complete soft tissue healing, a radiolucent lesion was evident at a later time, a recurrence was recorded.

Untreated cases had follow-up evaluations to assess for development of new, previously absent, cystic lesions (defined as incipient cysts), progression of pre-existing lesions (progressive cyst), or if the clinical and radiographic appearance of the unerupted tooth with or without a previously diagnosed cyst was unchanged (defined as static or no cyst, respectively).

Finally, it was evaluated if the size of coronal radiolucencies present around unerupted teeth could predict the nature of the lesions (i.e., dentigerous cyst vs. dental follicle), as recently suggested (14). In particular, it was assessed if pathological cystic lesions always appeared larger than three times the width of the periodontal space of a normal canine tooth in the same patient (14).

### Statistical Analysis

Descriptive analyses were performed to characterize the population of dogs and cats if at least five cases per group were present. Contingency tables were used to verify if the type of teeth was associated with the presence of a cyst. Chi^2^ test was used to identify an association between the type of inclusion of unerupted teeth and the presence of cysts. The above tests were also used to identify an association between the type of inclination of unerupted teeth and the presence of cysts, and to compare the frequency of unerupted teeth between types of teeth. The overall number of first, second and third incisor, canine, first and fourth premolar teeth was considered to be 276, assuming that all of the listed teeth were present in the 69 dogs (i.e., 69 × 4 = 276); the overall number of third molar teeth was considered to be 138, assuming that each of the 69 dogs had 2 third molar teeth (i.e., 69 × 2 = 138). To evaluate differences between type of most frequent unerupted teeth (i.e., canine, first premolar, and third molar) and body weight, the Kruskal–Wallis test was applied. To compare the age of dogs with and without dentigerous cysts, the Mann–Whitney test was applied. Significance was considered for *P* < 0.05.

## Results

Seventy-three cases [69 (94.5%) dogs and 4 (5.5%) cats] with a total of 113 unerupted teeth were included in the study (Supplemental Table 1).

### Signalment

Three cats were European short hair and one was a Maine coon. There were one female and three male cats, all neutered. The ages at the time of diagnosis were 1, 2.5, 5, and 10 years, respectively. The body weight was 4, 5 (2 cats), and 8 kg.

Canine breeds included boxer [20 dogs (28.9%)], Labrador retriever [6 dogs (8.7%)], Maltese, Chihuahua and mix breed [5 dogs each (7.2%)], pugs [4 dogs (5.8%)], Bernese mountain dog, Yorkshire terrier, Shih-tzu, spitz, and miniature schnauzer [2 dogs each (2.9%)], bull terrier, bull mastiff, Czechoslovakian wolfdog, English setter, Epagneul papillon, French bulldog, German shepherd, golden retriever, Irish setter, Italian shepherd, miniature poodle, Pekingese dog, pinscher, pitbull [1 dog each (1.4%)].

Forty-one dogs (59.4%) were males (38 intact and 3 neutered) and 28 (40.6%) females (13 intact and 15 neutered).

The mean and median ages at the time of diagnosis in dogs was 4.4 and 3.6 years, respectively (range from 5 months to 10.5 years).

The mean body weight for dogs was 21.0 kg (median 25 kg, range from 1.8 to 59.5 kg).

### Chief Complaint

The main reasons for dental consultation included the presence of an oral swelling/mass at or near the area of a missing (unerupted) tooth [8 cases, including 1 cat (10.9%)], which could be due to a cystic or neoplastic development, a missing tooth that was in fact unerupted [7 cases (9.6%], and discoloration of a tooth near the area of a missing (unerupted) tooth [1 case (1.4%)]. The remaining 57 cases (78.1%), including three cats, presented for unrelated reasons (e.g., periodontal disease, palatal defect, tooth fracture) and the presence of the unerupted tooth/teeth was an incidental finding diagnosed during the routine intraoral radiographic examination.

### Number and Type of Unerupted Teeth

**Figure 2:**
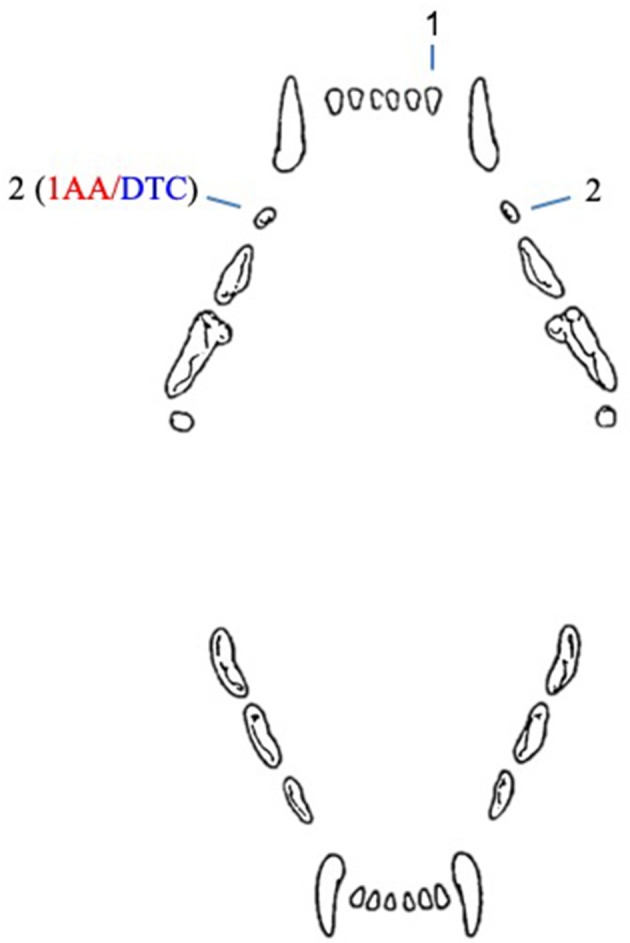
Number and type of unerupted teeth in cats included in the study. AA, associated ameloblastoma; DTC, teeth with radiographically or histologically confirmed dentigerous cyst.

In cats, five unerupted teeth were diagnosed in four patients (Figure 2). In one case, both maxillary second premolar teeth were unerupted.

**Figure 3:**
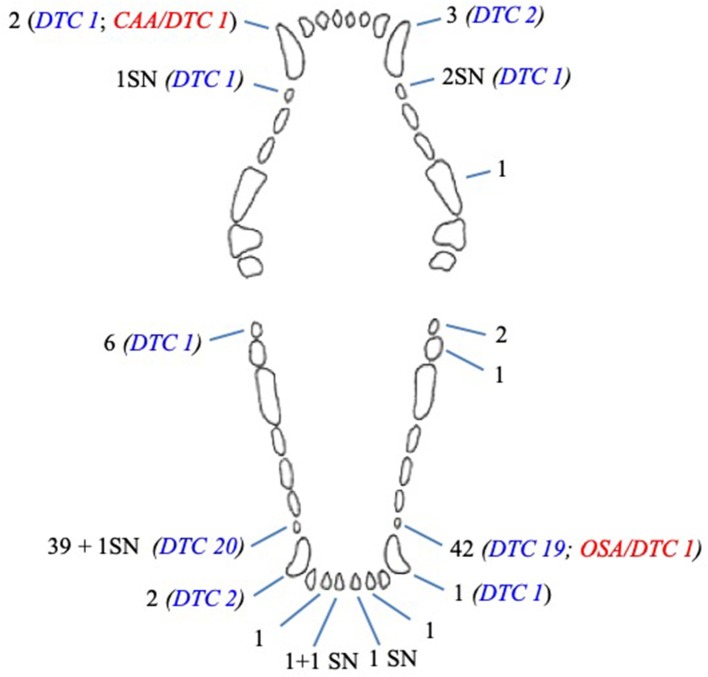
Number and type of unerupted teeth in dogs included in the study. CAA, associated canine acanthomatous ameloblastoma; DTC, teeth with radiographically or histologically confirmed dentigerous cyst; OSA, associated osteosarcoma; SN, supernumerary tooth.

In dogs, 108 unerupted teeth were diagnosed: 85 (78.7%) first premolar teeth, 8 (7.4%) canine teeth, 8 (7.4%) mandibular third molar teeth, 5 (4.6%) mandibular incisor teeth, 1 (0.9%) mandibular second molar tooth, and 1 (0.9%) maxillary fourth premolar tooth (Figure 3).

Multiple unerupted teeth were diagnosed in 29 (42%) dogs (Table 1).

**Table 1:** Multiple unerupted teeth (T/I) in dogs (numbering based on the modified Triadan system).

**Total number of dogs with multiple T/I**	**Total number of T/I**	**Number of dogs and location of T/I**
2	4	1: 105SN-205SN-305-405
		1: 305-311-405-411
5	3	1: 304-305-404
		1: 305-311-405
		1: 104-204-405
		1: 301-301SN-401SN
		1: 205SN-305-405
22	2	19: 305-405
		1: 204-305
		1: 208-411
		1: 311-411

*SN, supernumerary tooth*.

Lack of eruption was more frequent for first premolar teeth compared to all other teeth (*p* < 0.0001, for each contrast), and for third molar teeth compared to second molar teeth (*p* = 0.0009), fourth premolar teeth (*p* = 0.0009), second incisor teeth (*p* = 0.0031), and first incisor teeth (*p* = 0.008) (Table 2).

**Table 2:** Comparison of the frequency of unerupted teeth in dogs.

**UT**	**PM1**	**M3**	**M2**	**I1**	**I2**
PM1		*p* < 0.0001^*^	*p* < 0.0001^*^	*p* < 0.0001^*^	*p* < 0.0001^*^
M3	*p* < 0.0001^*^		0.0009✓	*p* = 0.008✓	*p* = 0.0031✓
M2	*p* < 0.0001^*^	*p* = 0.0009✓		NS	NS
I1	*p* < 0.0001^*^	*p* = 0.008✓	NS		NS
I2	*p* < 0.0001^*^	*p* = 0.0031✓	NS	NS	
PM4	*p* < 0.0001^*^	*p* = 0.0009✓	NS	NS	NS

UT, unerupted tooth;

* *statistically significant for PM1; ✓ statistically significant for M3; NS, not significant. Statistical test: r × c contingency table*.

The median body weight of dogs demonstrating unerupted canine, first premolar and third molar teeth was 3 (min 2.6–max 18), 28 (min 1.8–max 35), and 5.4 (min 2.7–max 9.6) kg, respectively. Body weight of dogs with unerupted first premolar teeth was greater than in dogs with unerupted canine and third molars (*p* = 0.005 and *p* = 0.004, respectively).

### Neoplastic Lesions and Dentigerous Cysts

A 10-year-old, neutered male, 7.8 kg, domestic short hair cat presented with an unerupted right maxillary second premolar tooth associated with a cystic lesion and an ameloblastoma. This was the only cystic lesion diagnosed in cats. The other unerupted teeth in cats were not evaluated histologically, but appeared radiographically negative for cystic development.

Concomitant neoplasias and cystic lesions were also histologically diagnosed in two dogs with unerupted teeth: a 5 months old, intact male, English setter dog with a canine acanthomatous ameloblastoma (CAA) (associated with an unerupted right maxillary canine tooth) and a 9 years old, female spayed, Boxer dog with an osteosarcoma (associated with an unerupted left mandibular first premolar tooth). Because of the impossibility to determine if the neoplastic and cystic lesions developed independently or were related changes, these teeth were not considered in the following analysis of cases with cysts.

Therefore, apart from these oncological cases, the presence of a dentigerous cyst was diagnosed in association with 48 teeth (44.4% of the unerupted teeth in dogs) in 34 canine patients, by radiography, and/or histology (Supplemental Table 1).

Thirty-four cysts (77.3%) developed in 25 brachycephalic dogs (boxer, pug, Maltese, Shih-tzu, and Chihuahua dogs), and 14 cysts (22.7%) in nine non-brachycephalic dogs (Labrador retriever, miniature pinscher, Italian shepherd, Bull terrier, Czechoslovakian wolf dog, and German shepherd dogs).

### Radiographic vs. Histological Diagnosis of Dentigerous Cyst in Dogs

In dogs, full-mouth radiographs were available for 66 cases, and partial radiographic examination (including radiographs of all edentulous areas) for remaining three cases.

Excluding the cases with neoplastic disease, a well-defined radiolucency compatible with a dentigerous cyst was radiographically evident for 44 teeth. The diagnosis was histologically confirmed in 34 (77.3%) of these cases, while in the remaining 10 cases histology was not performed.

Four teeth were radiographically equivocal (two teeth) or not evident (two teeth), however cystic lesions were confirmed histologically.

The presence of a dentigerous cyst was suspected for 19 other teeth, but the radiolucency was too small to reach a definitive radiographic diagnosis and histology was not performed.

Finally, the radiographic examination was considered negative for cystic changes in the remaining 39 unerupted teeth. Only six of these cases were evaluated histologically, confirming the absence of cystic tissues.

Six of eight (75%) unerupted canine teeth and 41 of 85 (48.8%) unerupted first premolar teeth developed a radiographic and/or histological change confirming a dentigerous cyst (Figure 3). The remaining cystic lesion was associated with a mandibular third molar tooth. There was a statistical association between the type of teeth and the absence of cyst (*p* = 0.010); in particular, the mandibular third molar tooth associated with the absence of cyst (*p* = 0.038). No statistical association was documented between the type of teeth and the presence of cyst, although a trend was observed for canine and first premolar teeth (*p* = 0.089 and *p* = 0.094, respectively).

### Characteristics of Dogs Showing Dentigerous Cyst

Dentigerous cysts were diagnosed in 18 of 20 (90%) boxer dogs (25 cysts), 2 of 6 (33.3%) Labrador retrievers (2 cysts); 2 of 5 Chihuahuas (3 cysts) and Maltese dogs (5 cysts); 2 of 4 pugs (4 cysts), and mix breed dogs (2 cysts); and 1 of 2 Shih-tzu dogs (2 cysts). Each of the single Italian shepherd, German shepherd, bull terrier, Czechoslovakian wolfdog, and pinscher dogs developed one cyst each.

Twenty-nine of 48 dentigerous cysts (60.4%) were diagnosed in intact male dogs, 6 (12.5%) in neutered male dogs, 5 (10.4%) in intact female dogs, and 8 (16.7%) in female spayed dogs.

The mean age at the time of diagnosis of the dogs showing dentigerous cysts was 4 years (median 3.3 years; range from 1.2 to 9.2 years).

The mean body weight of dogs with dentigerous cyst was 26.1 kg (median 30.8 kg; range from 3 to 46 kg).

### Histopathologic Findings

Histological examination was performed on excised tissues associated with 47 (40.4%) unerupted teeth (one from a cat and 46 from dogs), including the three neoplastic cases.

Submitted histological samples included: the lining of the cystic lesion only (nine teeth), the lining of the cystic lesion and the extracted teeth (29 teeth), soft tissue (i.e., gingiva) covering the crown of the unerupted teeth (when operculectomy was performed) (three teeth), gingiva and lining of the cyst (two teeth), the unerupted tooth only (one tooth), an incisional biopsy of a suspected neoplastic lesion associated with an unerupted tooth (one tooth), or the surgical excised maxillary portion including the neoplastic lesion and the unerupted tooth (two cases, of which one was a cat).

**Figure 4:**
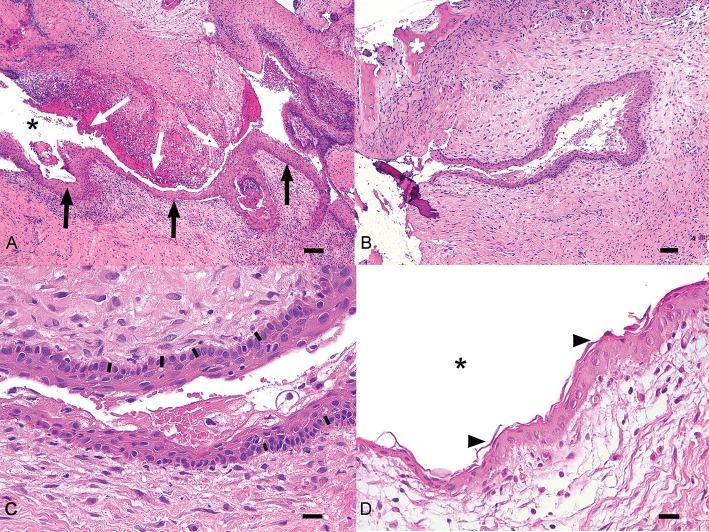
Histologic representation of a typical dentigerous cyst from one of the canine cases included in the study. **(A)** The wall of the cyst is lined by multistratified epithelium (black arrows) surrounded by moderate fibrous stroma with multifocal chronic infiltrates. In some areas the epithelium is detached (white arrows). Black asterisk, lumen of the cyst. Bar = 100 μm. **(B)** An irregular area of cementum (white asterisk) is visible in the stroma near the cyst. Bar = 100 μm. **(C)** In this field, the epithelium shows a basilar palisade similar to odontogenic epithelium (black bars). Bar = 25 μm. **(D)** The epithelium is characterized by parakeratotic keratosis (arrowheads). Black asterisk, lumen of the cyst. Bar = 25 μm.

Three cases presented concomitant cystic and neoplastic diseases associated with unerupted teeth. One cat had an ameloblastoma, one dog had a CAA and one dog an osteosarcoma. Histologic diagnosis of dentigerous cyst was made for 38 other teeth. All samples showed histological similarities. The cystic lesions were often multilocular, lined by a variably thick, multilayered, squamous epithelium with multifocal or diffuse keratinization and multifocal parakeratosis (Figure 4). Keratin was scarce or moderate, never filling the lumen. Often the stratum basale was described as palisading. Occasionally, apoptosis and superficial cytoplasmic vacuolar degeneration were present. The epithelium was often multifocally detached, with hemorrhage and fibrin deposition. The wall was composed of dense fibrous tissue frequently showing a multifocal, mild to severe, mononucleated infiltrate (lymphocytes, plasma cells, macrophages, hemosiderophages), and sometimes cholesterine crystals (a sign of cellular damage). Neutrophils were sometimes present. Multifocal, small irregular areas of newly formed cementum/osseous tissue were also a common finding.

Six of 47 histological samples were negative for cystic or neoplastic development.

### Differentiation Between Small Dentigerous Cysts and Normal Dental Follicle

Ten of the 62 teeth that were either radiographically equivocal or negative for the presence of cystic lesions were evaluated histologically (Table 3).

**Table 3:** Differential diagnosis between small dentigerous cysts and normal dental follicle.

**Breed**	**Age (months)**	**Weight (Kg)**	**T/I**	**Pericoronal space (mm)**	**PDL space of canine tooth (mm)**	**Pericoronal space: PDL space of canine tooth ratio**	**Rx**	**Histology**
Mix breed	84	25	405	0.9	0.5	1.8	Dubious	Cyst
Bull terrier	19	18	204	0.9	0.6^*^	1.5	No cyst	Cyst
Maltese dog	19	5.4	305	0.8	0.4	2	Dubious	Cyst
Pinscher	36	4	405	0.3	0.3	0	No cyst	Cyst
Mix breed	92	11	305	0.8	0.2	4	No cyst	No cyst
Miniature poodle	8	5.8	305	0.7	0.2	3.5	No cyst	No cyst
Miniature poodle	84	5.8	405	0.6	0.2	3	No cyst	No cyst
Boxer	54	46	305	0.5	0.2	2.5	No cyst	No cyst
Chihuahua	18	2	405	0.3	0.3	0	No cyst	No cyst
Chihuahua	18	2	305	0.3	0.3	0	No cyst	No cyst

Width of pericoronal radioluncency and periodontal ligament space of a reference tooth [the adjacent or contralateral (

* *) canine tooth] for radiographically dubious or negative teeth evaluated histologically. Numbering based on the modified Triadan system. Rx, radiographic diagnosis for cystic lesion; T/I, unerupted tooth*.

Four of these samples histologically demonstrated evidence of a dentigerous cyst, even though the size of their pericoronal radiolucency was equal or less than three times the width of the periodontal ligament space of the reference tooth.

Three teeth histologically consistent with dentigerous cysts demonstrated a pericoronal radiolucency that was in fact three times or more the width of the periodontal ligament space of the adjacent canine tooth.

The histological examination of the remaining three teeth (two with pericoronal radiolucency and periodontal ligament space of the reference tooth of identical dimensions, one with a pericoronal radiolucency that was 2.5 times the size of the periodontal ligament space of the referenced tooth) were negative for histologic signs consistent with a cyst.

### Depth of Inclusion of Unerupted Teeth

In feline patients, one of five unerupted teeth was in complete bone inclusion, and four teeth were in soft tissue inclusion, including one tooth associated with an ameloblastoma (Table 4).

**Table 4:** Inclination and type of inclusion of unerupted teeth with and without confirmed dentigerous cyst in cats.

	**Tooth inclination**										**Type of inclusion**			
	**MA**		**DA**		**V**		**H**		**I**		**Bone inclusion**		**Soft tissue inclusion**	
	**With DTC**	**Without DTC**	**With DTC**	**Without DTC**	**With DTC**	**Without DTC**	**With DTC**	**Without DTC**	**With DTC**	**Without DTC**	**With DTC**	**Without DTC**	**With DTC**	**Without DTC**
203	0	0	0	0	0	1	0	0	0	0	0	1	0	0
106–206	0	0	0	0	1^*^	3	0	0	0	0	0	0	1^*^	3
**Total**	**0**	**0**	**0**	**0**	**1**	**4**	**0**	**0**	**0**	**0**	**0**	**1**	**1**	**3**

Numbering based on the modified Triadan system. DA, distoangular; H, horizontal; MA, mesioangular; V, vertical; I, inverted; DTC, dentigerous cyst;

* *tooth associated with cyst and neoplasia*.

In dogs, 25 (23.6%) of 106 unerupted teeth were in complete bone inclusion (10 teeth with evidence of cystic development), and 81 teeth (76.4%) were in soft tissue inclusion at the time of diagnosis (38 teeth with cystic development) (Table 5). The remaining two teeth, associated with neoplastic lesions, were also considered in soft tissue inclusion.

**Table 5:** Inclination and type of inclusion of unerupted teeth with and without confirmed dentigerous cyst in dogs.

	**Tooth inclination**										**Type of inclusion**			
	**MA**		**DA**		**V**		**H**		**I**		**Bone inclusion**		**Soft tissue inclusion**	
	**With DTC**	**Without DTC**	**With DTC**	**Without DTC**	**With DTC**	**Without DTC**	**With DTC**	**Without DTC**	**With DTC**	**Without DTC**	**With DTC**	**Without DTC**	**With DTC**	**Without DTC**
301–401	0	0	0	0	0	1	0	0	0	2	0	0	0	3
302–402	0	0	0	0	0	0	0	2	0	0	0	1	0	1
104–204	0	0	0	0	0	0	4^*^	1	0	0	1	0	3^*^	1
208	0	0	0	0	0	1	0	0	0	0	0	0	0	1
304–404	0	0	0	0	0	0	3	0	0	0	0	0	3	0
105–205	0	0	0	0	0	0	2	1	0	0	0	0	2	1
305–405	10	20	1	0	13^*^	8	12	13	4	0	9^*^	11	31	31
310–410	0	0	0	0	0	0	0	2	0	0	0	2	0	0
311–411	0	0	0	0	0	4	1	2	0	1	0	1	0	6
**Total**	**10**	**20**	**1**	**0**	**13**	**14**	**22**	**21**	**4**	**3**	**10**	**15**	**39**	**44**

Numbering based on the modified Triadan system. DA, distoangular; H, horizontal; MA, mesioangular; V, vertical; I, inverted; DTC, dentigerous cyst;

* *tooth associated with cyst and neoplasia*.

No statistical association was found between the type of inclusion and the presence of cysts (*p* = 0.686).

### Inclination of Unerupted Teeth

All five unerupted feline teeth were in vertical inclination, including the tooth associated with the ameloblastoma (Table 4).

In dogs, 43 (39.8%) teeth were positioned horizontally, 31 were angled [30 (27.8%) in mesioangular and 1 (0.9%) in distoangular direction], 27 (25%) were in vertical position, and 7 (6.5%) were inverted (Table 5). The remaining two teeth, associated with neoplastic lesions, were in vertical and horizontal inclination. There was no statistical difference between the teeth in vertical inclination and the teeth with a different inclination, and cystic development (*p* = 0.879).

The radiographic inclination of unerupted teeth that developed dentigerous cysts was horizontal in 21 cases (43.8% of teeth with dentigerous cysts; 51.2% of teeth in horizontal position), vertical in 12 cases (25% of teeth with dentigerous cysts; 44.4% of teeth in vertical position), mesioangular in 10 cases (20.8% of teeth with dentigerous cysts; 33.3% of teeth in mesioangular position), and inverted in four cases (8.3% of teeth with dentigerous cysts; 57.1% of teeth in inverted inclination); distoangular in one case (2.1% of the teeth with dentigerous cysts).

There was no statistical association found between the type of inclination and the presence of cysts (*p* = 0.484).

### Abnormalities Affecting Unerupted and Adjacent Teeth

External resorption of the canine tooth adjacent to the neoplastic lesion was evident in the cat with ameloblastoma associated with an unerupted right maxillary second premolar tooth.

Thirty-six dogs demonstrated abnormal unerupted and/or adjacent teeth. External root resorption was commonly associated with large cystic lesions or tumors (42 teeth). Four unerupted teeth were malformed. One dog was affected by generalized dentin dysplasia, also involving the unerupted tooth. Three teeth that were secondarily involved by the expanding cystic lesion were non vital. Three teeth adjacent to unerupted teeth were missing and 15 teeth showed morphological abnormalities (i.e., fused roots).

Thirty-three dogs did not have evidence of morphological or structural abnormalities of unerupted or adjacent teeth.

### Treatment

In cats, two unerupted teeth were extracted from the same patient, and two unerupted teeth (with no signs of associated complications) were left untreated, with the recommendation to radiographically monitor it over time. Partial maxillectomy including the unerupted tooth was performed for treatment of the ameloblastoma.

In dogs, operculectomy was performed to treat seven (6.5% of unerupted teeth) first premolar teeth, one of which showed radiographic and histological signs of cystic development. Forty-seven (43.5%) teeth without signs of dentigerous cyst were simply extracted, 38 (35.2%) teeth were extracted and curettage of the cystic lining was performed, and one (0.9%) tooth was excised during a rostral maxillectomy performed to treat a CAA. The remaining 15 (13.9%) unerupted teeth (four with radiographic signs of cystic development and one associated with an osteosarcoma) were not treated. The large OSA was treated by palliative intralesional excision and radiation therapy.

### Outcome of Treatment

In cats, clinically complete soft tissue healing was evident at the last follow-up visit for three teeth (two extracted teeth, 1 month follow-up; one tooth included in a maxillectomy procedure, 2 months follow-up). The clinical and radiographic re-examinations on two untreated teeth, performed in other two cats 3 months and a 3 years following diagnosis, showed no changes on the affected tooth or nearby bone or teeth.

In dogs, only clinical (43 teeth), clinical and radiographic (35 teeth), or clinical and tomographic (one tooth) follow-ups were available. Follow-ups were not available for the remaining 29 teeth.

Follow-up visits were performed from 1 to 68 months (mean 8 months; median 1.5 months) following diagnosis and/or treatment.

**Figure 5:**
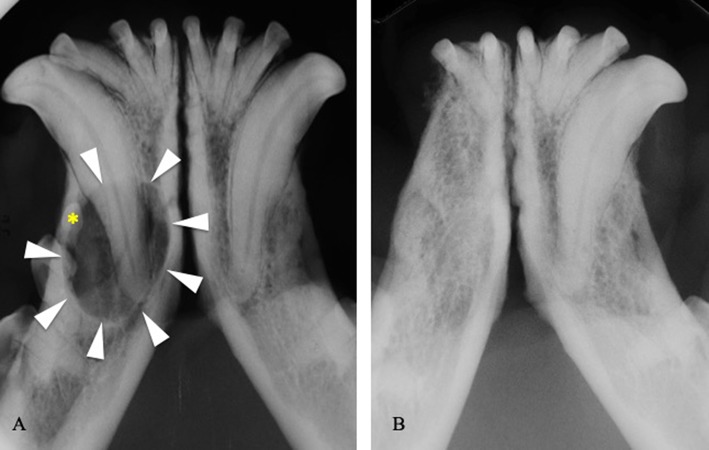
**(A)** Intraoral radiograph showing a dentigerous cyst (arrowheads) developed around the right mandibular first premolar tooth (asterisk) in a 3-years-old boxer dog. **(B)** Intraoral radiograph at the 1-year follow-up visit, following extraction of the right third incisor, canine, first and second premolar teeth, and cystic lining curettage.

In 62 of 85 cases treated by extraction and/or curettage, treatment appeared successful with complete healing of soft tissues and (in cases where diagnostic imaging was performed) new bone formation at the surgical site (100%) (Figure 5). No cases of recurrence were reported. The remaining 23 extracted teeth were lost to follow up.

Short-term follow-up re-examination was available for four teeth that were treated by operculectomy: a 1-month clinical re-examination in two cases and 3-month clinical and radiographic re-examination in other two cases. The procedure appeared successful (the crown of the treated tooth being still exposed above the gingival margin at follow-up), however further eruption was not apparent.

In the dog with an osteosarcoma associated with an unerupted left mandibular first premolar tooth, the untreated dentigerous cyst associated with its right mandibular first premolar tooth showed progression at the 3-month radiographic follow-up, but treatment was again refused by the owner. Another untreated cyst associated with a mandibular first premolar tooth showed progressive changes at the 6-month follow-up, and surgical excision was then performed. All other nine untreated unerupted teeth did not show clinical evidence of an incipient cyst, but none was radiographically re-examined.

The cases with the remaining four untreated unerupted teeth (two with dentigerous cyst) were lost at follow-up.

The 12-months follow-up of the dog with OSA showed significant radiographic enlargement of the neoplasia and the associated cystic lesion, despite palliative treatment. The maxillectomy site of the dog with CAA at the 1-month follow-up appeared clinically healed.

## Discussion and Conclusions

Tooth eruption is the axial movement of the tooth from its non-functional position in the bone to functional occlusion (17). It is a multifactorial, not yet fully understood process, starting from the reduced enamel epithelium (REE) covering the crown of the developing tooth, which fuses with the gingiva during the process. The REE produces proteases that, by breaking down connective tissue, decrease the tissue resistance to eruption. The dental follicle is also essential in the creation of an eruptive pathway for the tooth by modulating bone remodeling. It produces growth factors promoting the differentiation of monocytes into osteoclasts, that create a path to eruption. At the same time the production of osteoblasts from mesenchymal stem cells is accentuated at the base of the alveolar crypt (17).

Teeth may fail to erupt because of mechanical obstruction by a physical barrier (e.g., gingiva, bone, neoplastic tissue, and tooth crowding) (defined as tooth impaction), or because of an impaired mechanism of eruption [e.g., a dysfunction of REE cells (18)] (defined as dental retention, or embedded teeth) (19). In humans a discrepancy between jaw and tooth size is described as a possible cause of the frequent impaction of the third molar teeth (5).

It is interesting to note that 6 of 8 unerupted canine teeth (in Maltese and Chihuahua dogs) and 7 of 8 unerupted mandibular third molar teeth (in Yorkshire terrier, Maltese, Shih-tzu, Epagneul papillon, and pug dogs) in the present study were diagnosed in small breed dogs. The reason could be related to the limited jaw size typical of these breeds, frequently also showing congenitally missing third molar and other teeth (20).

In agreement with the literature, in the present study it was also found that brachycephalic dogs were overrepresented (40 dogs; 57.9%). Boxer dogs in particular accounted for 28.9% of the canine study population. Interestingly, they only showed unerupted first premolar teeth, and 18 out of 20 boxer dogs developed at least one dentigerous cyst. Furthermore, the only three unerupted maxillary first premolar teeth (all being supernumerary teeth) were found in this breed. Another interesting finding was the high prevalence of dentigerous cyst formation in boxer dog (78% of unerupted teeth in this breed developed a cyst, and overall 52% of cysts associated with unerupted teeth were diagnosed in boxers).

Moreover, in the entire study period a total of 39 boxer dogs were anesthetized and evaluated radiographically by the dental and oral surgery departments of the involved veterinary clinics (data not shown). Therefore, it is notable that more than 50% of boxers presented for various dental and oral problems had at least one unerupted tooth (20 dogs), and that most of these developed associated lesions (18 dentigerous cysts and 1 OSA).

Brachycephalic dogs often show tooth rotation, tooth crowding, and reduced interproximal spaces. However, in the cases described here most unerupted teeth were located at normally wide interproximal spaces. Also, the gingival tissues appeared clinically normal in consistency and thickness, the alveolar bone did not seem abnormally dense, either radiographically or during extraction, and only in a few cases dental crowding (six unerupted teeth being supernumerary) or the presence of neoplastic tissues over the affected teeth could justify a mechanical obstruction to eruption. Therefore, other pathogenetic mechanisms may have been involved. Histologically normal gingival tissues overlying numerous impacted teeth were described in a 7-month-old Bedlington terrier dog (21).

A genetic basis is hypothesized for the primary failure of eruption (PFE) in humans. PFE is a non-syndromic condition that occurs without any obvious evidence of obstruction, most commonly affecting non-succedaneous teeth (i.e., the molar teeth) (22). This condition seems to be linked to a mutation of the PTH1R (parathyroid hormone receptor 1) gene that, in association with the parathyroid like hormone, seems to be able to affect number, quality and function of osteoclasts and osteoblasts (23, 24). Reduced bone resorption by osteoclast cells and bone formation by odontoblasts could contribute to dental eruption failure (23). Genetic studies would be necessary to evaluate if a similar mechanism could be involved in particular for failure of eruption of the first premolar and molar teeth (non-succedaneous teeth) in dogs and if a genetic breed predisposition exists. In the present study all cases with multiple unerupted teeth showed lack of eruption in the same jaw (mandibles or maxillae), or unilaterally and isolated to one side. Only one of 29 cases demonstrated unerupted teeth in diagonally opposite jaws (left maxillary fourth premolar tooth and right mandibular third molar tooth). These findings may support the potential for an underlying genetic influence contributing to the condition.

One of the important limitations of the present study is the lack of data of the general population visited during the same period of time at the same veterinary clinics. It is therefore difficult to demonstrate definite associations between lack of eruption and development of associated lesions (i.e., cysts and tumors), and breed, age, body weight, sex, and neutering status of the animals.

However, based on data provided by the Italian kennel club (ENCI) on dogs registered in Italy in the last 5 years, the distribution ratio between boxers and Labrador retrievers in the country is greatly in favor of the latter breed (1 boxer: 2.6 Labrador retrievers) (ENCI)^1^. Therefore, the present findings (few unerupted teeth in a few Labrador retrievers, despite their wide distribution in the country, and numerous unerupted teeth in a relatively large number of boxer dogs, in spite of their limited distribution) may support a genetic predisposition in boxers.

Similarly to the cases published in the veterinary literature (13, 14, 20, 21, 25–61), the first premolar tooth was by far the most frequently unerupted tooth, followed by the maxillary/mandibular canine teeth. In our study population, the mandibular third molar tooth was also frequently affected.

Also, the great majority of unerupted teeth in dogs in this study were mandibular teeth (91%), similarly to the epidemiologic data reported in humans (4, 62, 63).

Tooth inclination and depth of inclusion into hard or soft tissues in human patients are important features used by surgeons to predict the degree of difficulty of surgical extraction of unerupted teeth (15). Seventy-five percentage of impacted mandibular third molar teeth are reported in mesioangular and horizontal inclination, but the inclination of those developing into dentigerous cysts is mainly vertical (64%) or horizontal (41%) (64, 65).

In the present study, unerupted teeth in dogs were also mainly in horizontal (41%) and mesioangular (30%) inclination, with a total of 75.9% of teeth in a position different from the physiologic (vertical) inclination. Considering how the tooth germs normally develop and erupt following an axial path, it could therefore be speculated that tooth inclination may represent a possible predisposing factor to eruption failure (66, 67). However, tooth inclination at the time of diagnosis may reflect the original (abnormal) position of the tooth germ (i.e., representing a cause for eruption failure) or be a result of an attempt of eruption against a physical barrier (e.g., gingiva or bone) forcing the tooth in a different position. Therefore, this hypothesis remains to be elucidated.

The development of dentigerous cysts is commonly associated with unerupted teeth in humans and animals (68, 69). Several veterinary case reports (21, 25, 28–30, 32–34, 37, 42, 50, 52–54, 57, 60, 70) and a few retrospective studies (13, 14, 20) describe dentigerous cysts associated with unerupted teeth in dogs. Cysts may develop by accumulation of fluid between the REE and enamel, or between the layers of enamel epithelium itself, but the exact pathogenetic mechanism remains unclear (71). Inflammation affecting a deciduous tooth has also been involved in the development of dentigerous cysts around the corresponding unerupted permanent teeth and in causing a volume increase of the cyst by exudate formation in humans (71–73). None of the cases reported here had history of trauma or other lesions affecting the deciduous dentition, but they can not be completely excluded.

In humans, the prevalence of DTC associated to unerupted third molar teeth (the most commonly unerupted teeth) is low, ranging from 1.4 to 2.31% (74, 75). Babbit et al. reported a 29.1% prevalence (62 teeth) for dentigerous cysts development in a selected canine population of 136 patients with unerupted teeth (14). A radiographic study performed in 233 small breed dogs to determine the prevalence of dental abnormalities described 25 unerupted teeth (0.3% of the total number of existing teeth) with a 32% prevalence of cystic development (8 teeth, the 0.1% of the total number of existing teeth) (20). In this publication all dentigerous cysts were diagnosed associated with mandibular first premolar teeth in Shih-tzu dogs (20). In the present study, a higher prevalence (44.4% dentigerous cysts formation) was reported. This difference could be related to the study methodology (i.e., diagnosis based on radiographic as well as histologic findings), and possibly to epidemiologic factors (e.g., possible predisposition in certain breeds, that may be more common in Italy as compared to the USA or Korea).

Most teeth (76.4%) were found in soft tissue inclusion, and almost half of these developed dentigerous cysts. However, cystic lesions enlarge over time, causing progressive bone resorption. Therefore, these teeth may have originally been in bone inclusion, and it can not be determined if depth of inclusion is actually a predisposing factor to cyst development.

Also, there was no statistical association between inclination angle and the presence of cystic lesions.

In the present study the mandibular first premolar tooth was not only the most commonly diagnosed unerupted tooth (85 of 108 unerupted teeth), but was also most commonly associated with cyst development (41 of 48 dentigerous cysts). This is also in agreement with the data extrapolated from the literature, with cysts described in association with 49 out of 71 (69%) reported unerupted first premolar teeth (13, 14, 20, 29, 50, 54, 57, 58, 60, 70). Interestingly, six of eight unerupted canine teeth in the present study were associated with a cystic lesion, one with a neoplastic lesion, and one showed a radiographically equivocal pericoronal appearance. However, only one of eight unerupted third molar teeth had a radiographically evident and histologically confirmed cyst, and another one had a radiographically equivocal appearance. Even though it could be speculated that the high prevalence of cyst formation at the canine teeth as compared to mandibular third molar teeth could be related to the greater REE surface of larger teeth, the high prevalence of cysts associated with first premolar teeth would contradict this theory.

Typical histological characteristics of dentigerous cysts associated with unerupted teeth were repetitively observed. However, the diagnosis is sometimes challenging, particularly in case of small biopsy samples, or because of the cystic nature of the tissues, that can be difficult to sample or may be incorrectly oriented. Also, the lining epithelium may be partially lost from the samples. In these cases, it is important to look for and evaluate other typical characteristics, like the presence of a cystic space combined with the presence of fibrin or cholesterin crystals, keratin, and/or cementum/osseous tissue in a fibrotic wall, and/or a chronic infiltrate. Finally, as always, histopathological findings should be correlated to the radiological and clinical presentation.

In humans, tumors may be associated with unerupted teeth with or without dentigerous cysts (75, 76). The epithelial component seems to be essential for neoplastic transformation, but the exact pathogenetic mechanism is still unclear (74, 75). Odontomas are among the most common associated tumors (77, 78), and are mainly diagnosed in the second decade of life (77, 79). In about one fourth of these patients, dentigerous cysts are also present (76). Surgical excision of the tumor and extraction of the associated unerupted teeth is the recommended treatment, however tumor excision and denudation of the unerupted teeth, followed by orthodontic treatment, is sometimes possible (78).

The risk of developing tumors within the wall of a dentigerous cyst in humans is rare (6, 75). Ameloblastomas, ameloblastic fibromas, fibro-odontomas and fibrosarcomas, adenomatoid odontogenic tumors, keratocystic odontogenic tumors, calcifying epithelial odontogenic tumors, mucoepidermoid carcinomas, odontogenic carcinomas, and squamous cell carcinomas have been described associated with dentigerous cyst, with a 0.14–2% incidence (3, 6, 74, 80–82). In these cases, chronic inflammation, keratin metaplasia or the continuous intracystic pressure are suggested as possible causes of tumor development (83–85).

Neoplastic lesions associated with unerupted teeth have previously been described in 16 dogs: 13 odontomas (7 compound odontomas, 1 ameloblastic odontoma, 1 ameloblastic fibro-odontoma, and 4 non-specified odontomas), 1 squamous papilloma, 1 peripheral odontogenic fibroma, and 1 squamous cell carcinoma (14, 26, 31, 32, 38, 39, 44, 47, 52, 56, 59, 86). Only in six out of 16 cases (37.5%) a cystic component was also described (32, 39, 59, 86). The dogs with reported age were all very young (<12 months), except for a 4 years old dog with a cystic peripheral odontogenic fibroma. When reported, the treatment involved surgical excision of the tumor, including the unerupted tooth.

In our study, two ameloblastomas and one osteosarcoma were diagnosed closely associated with unerupted teeth. In all three cases cystic lesions were also present. In the 5 month-old dog, lack of eruption could have been secondary to the presence of the neoplastic tissue, with secondary cyst formation. On the other hand, canine ameloblastoma is an epithelial tumor of the adult age (87). Therefore, the development of this lesion in such a young patient may support the hypothesis of a direct origin from the DTC. In the other two cases the tumors developed at an older age, several years after tooth eruption should have taken place, and the presence of the neoplastic tissue was unlikely the cause of tooth impaction. Moreover, the possibility for the osteosarcoma, a mesenchymal tumor, to originate from the epithelial elements of a DTC is very low and the two lesions were likely independent entities.

Histologically, it is difficult or often impossible to determine if a neoplastic lesion is concomitant (but independent from) or if it is directly associated with the adjacent cyst. It is reported that if there is a direct connection between the wall of the cyst and an epithelial neoplasia, the latter arises from the cyst (85). However, to reveal a potential connection between the cyst and the tumor it would be necessary to histologically examine the entire cystic wall, which is rarely possible.

Unerupted teeth have rarely been reported in cats. A prevalence of 2.3% teeth was described in a study on value of full mouth radiography in 115 cats (88). Unerupted teeth (4 maxillary canine teeth and 1 maxillary third incisor tooth) were also reported in 5 of 50 brachycephalic cats in a prospective study on oro-dental anomalies in Persian and Exotic cats (12). Dentigerous cysts were not described in these studies. Two clinical cases described the development of dentigerous cysts associated with unerupted canine teeth (9, 10). One further case report described multiple unerupted teeth in a cat with abnormal eruption of the entire dentition, supposedly due to regional odontodysplasia (11). Retained and persistent deciduous (and permanent) teeth are also a relatively common feature in cats showing atraumatic patellar fracture, possibly caused by a still undefined primary bone disorder [the so called “Knees and Teeth Syndrome” (KaTS) or “Patellar Fracture and Dental Anomaly Syndrome” (PADS)], but these teeth do not seem to develop dentigerous cysts (89–91). Finally, three cases of feline inductive odontogenic tumors associated with unerupted canine teeth have been published (92–94). In the present study only an unerupted maxillary second premolar tooth developed a cystic lesion, which was histologically associated with an ameloblastoma and which exact origin could not be determined. The other four unerupted teeth in the remaining three cats did not show evidence of cystic development. However, given the extremely small feline study population, no conclusion can be drawn on incidence and possible predisposing factors to cystic development in this species.

In humans, a higher incidence of tooth impaction is reported in women (3, 4, 63). This could be explained by the smaller size of the jaws as compared to men (63). On the other hand, a male predisposition is described in developing dentigerous cysts associated with unerupted third molar teeth (68, 69). Sex and/or neuter status have not been reported to be associated with either lack of eruption or cystic development in veterinary patients, and this observation was confirmed by the present study. The relatively high number of intact male dogs reported is likely due to the uncommon habit of neutering male dogs in Italy.

The relatively older age of the animals at the time of diagnosis in this study may be explained by the fact that dogs and cats are rarely brought to veterinary attention because of a missing tooth (13). In fact, the main reason for presentation in this study was related to the unerupted teeth (i.e., clinical absence of a tooth, presence of an oral tumor or a visible cyst in an edentulous area) in only 22% of the cases. In most instances the diagnosis was incidental, following routine intraoral radiographic examination. Furthermore, it must be considered that even if a full dentition is clinically present, unerupted supernumerary teeth may be present. Therefore, this study highlights the usefulness of a complete radiographic examination in all dental patients, to obtain an accurate and early diagnosis. The use of more advanced diagnostic imaging modalities (CT or CBCT) may also be indicated to assess the presence of cystic lesions that may be too small to diagnose radiographically.

One of the main aims of this study was to try to elucidate if unerupted teeth should always be extracted or if conservative treatment is an acceptable approach in those cases that at the time of diagnosis do not show clinical or radiographic signs of pathology. In humans, indications for extraction are the presence of pathology of the dental follicle (cysts and tumors), recurrent pericoronitis, cellulitis, abscessation, resorption of adjacent roots or pain (95). As expected, in the present study the few cases initially diagnosed with dentigerous cysts but left untreated showed enlargement of the lesion at the follow-up visit performed up to 6 months after diagnosis. On the other hand, in two adult cats teeth that at the time of diagnosis were radiographically negative for cystic formation did not show any changes at the 3 months (1 tooth) and 3 years (1 tooth) follow-ups. In dogs, 9 teeth without radiographic evidence of dentigerous cyst were left untreated. However, they were only followed-up clinically rather than radiographically. Therefore, even if there was not clinical evidence of complications, these can not be completely excluded.

A large debate exists regarding the prophylactic extraction of asymptomatic unerupted third molars in humans, because of the high risk of iatrogenic injury to the inferior alveolar and lingual nerves, as well as the morbidity associated with the surgical procedure (1, 95). Coronectomy with intentional root retention is suggested in some of these cases (8). The guidelines by the American Association of Oral and Maxillofacial Surgeons (AAOMS) recommend the extraction of unerupted teeth showing evidence of or at risk of developing pathology, and strict clinical and radiographic control of untreated teeth (95). However, 50% of radiographically normal impacted teeth show histological evidence of cystic lining (64). In the present study, only 40.4% of unerupted teeth were submitted for histological evaluation, because of owners' preference or surgeon's recommendation (based on lack of radiographic changes). The prevalence of cystic development could therefore be higher than reported. In fact, histology revealed the presence of dentigerous cysts in two radiographically negative and two radiographically equivocal cases, based on the definition proposed by the available literature (14). Therefore, relying only on the radiographic examination to diagnose the presence of dentigerous cysts for the diagnosis may be misleading. Thus, extraction and histological evaluation of all unerupted teeth is recommended by the authors. The limited diagnostic yield of radiology and the risk of not treating asymptomatic teeth should also be discussed with the owners, and radiographic (or tomographic) follow-ups should be recommended in these cases.

The mean age of the dogs without clinical and radiographic evidence of cystic development was 4.8 years, which was statistically not significantly different from the animals with diagnosed dentigerous cysts (data not shown). Also, the age of the oldest dogs with and without cysts was very similar (9.2 and 10 years, respectively). Therefore, no conclusion can be drawn about the likelihood of cystic development based on age. However, it may be reasonable to consider radiographic surveillance of unerupted teeth diagnosed in older dogs if complications are not visible and extraction is considered complicated. Clear indications on appropriate follow-ups intervals are not available in the literature, but the authors in these cases recommend yearly re-checks. More frequent radiographic re-evaluations may be indicated in situations considered at higher risk (e.g., unerupted first premolar teeth in boxer dogs).

Few reports describe operculectomy as a treatment modality for unerupted teeth (21, 33). In the present study, the four cases treated this way and for which a follow-up re-examination was available showed a successful outcome. However, because of the small number of patients treated with this procedure and their short follow-up, definitive conclusions of the validity of this treatment can not be determined. Therefore, the risk of applying such non-standard approach should be discussed with the owners.

## Data Availability Statement

All datasets generated for this study are included in the article/Supplementary Material.

## Ethics Statement

This study reviewed data and treatments offered to client-owned dogs and cats accordingly to the current guidelines for treatment of cases affected by unerupted teeth. Each procedure was performed after obtaining an informed consent from the owners as indicated in the Legislative decree of 4 March 2014 No. 16, art. 2 and subsequent law No. 17710 from 26 July 2017 on non-experimental Veterinary Clinical Practices.

## Author Contributions

EB: collection and interpretation of data, and draft of the manuscript. MG: study design and critical revision of the manuscript. SF: evaluation and interpretation of histological samples and critical revision of histopathological part of the study. EZ: statistical analysis of the data.

### Conflict of Interest

The authors declare that the research was conducted in the absence of any commercial or financial relationships that could be construed as a potential conflict of interest.

## Footnotes

^1^http://www.enci.it/
