# Supplementary material for: A Clinical, Radiographic and Histological Study of Unerupted Teeth in Dogs and Cats: 73 Cases (2001–2018)
Source: Front Vet Sci. 2019 Nov 8;6:357. doi: 10.3389/fvets.2019.00357 (PMC6856145; doi:10.3389/fvets.2019.00357)
Supplement: Supplementary file 1 [file Data_Sheet_1.PDF]

Supplemental Table 1. Data of dogs and cats with unerupted teeth (T/I) included in the study. Numbering of teeth based on the modified Triadan system. BI: bone inclusion; CAA: canine acanthomatous ameloblastoma; Cl: clinical; CT: Computed Tomography; DSH: Domestic Shorthair cat; F: female; FS: female spayed; M: male; MC: male castrated; N: absent; NV: non vital tooth; OSA: osteosarcoma; Rx: radiographic; SN: supernumerary tooth; STI: soft tissue inclusion; TR: tooth resorption; Y: present; Y/N: dubious radiographic diagnosis of cystic lesion; DA: distoangular; H: horizontal; I: inverted; MA: mesioangular; V: vertical.

| Case N. | Lesion n. | Species | Breed | Age (months) | Sex | Weight (Kg) | T/I | Rx T/I position | Type of inclusion (BI/STI) | Cyst (Y/N) | Histology | Rx presence of cystic lesion | T/I treatment | Others T/I anomalies | Last follow up (months) | Follow-up | Clinical outcome |
|---|---|---|---|---|---|---|---|---|---|---|---|---|---|---|---|---|---|
| 1 | 1 | Cat | DSH | 61 | MC | 5.1 | 106 | V | STI | N | Not performed | N | T/I extraction | N | 2 | Cl | Healed |
| | 2 | Cat | DSH | 61 | MC | 5.1 | 206 | V | STI | N | Not performed | N | T/I extraction | N | 2 | Cl | Healed |
| 2 | | Cat | DSH | 13 | MC | 4.1 | 203 | V | BI | N | Not performed | N | None | N | 3 | Cl and Rx | No progression |
| 3 | | Cat | Maine Coon | 30 | FS | 5.2 | 206 | V | STI | N | Not performed | N | None | N | 41 | Cl and Rx | No progression |
| 4 | | Cat | DSH | 120 | MC | 7.8 | 106 | V | STI | Y | Ameloblastoma/ cyst | Y | T/I extraction and curettage | N | 1 | Cl | Healed |
| 1 | 1 | Dog | Pugs | 99 | M | 6 | 305 | H | BI | N | Not performed | Y/N | T/I extraction | N | None | None | / |
| | 2 | Dog | Pugs | 99 | M | 6 | 405 | H | BI | N | Not performed | Y/N | T/I extraction | TR 405 | None | None | / |
| 2 | 1 | Dog | Maltese | 42 | M | 3 | 304 | H | STI | Y | Odontogenic cyst | Y | T/I extraction and curettage | N | 12 | Cl and Rx | Healed |
| | 3 | Dog | Maltese | 42 | M | 3 | 305 | H | STI | Y | Odontogenic cyst | Y | T/I extraction and curettage | N | 12 | Cl and Rx | Healed |
| | 2 | Dog | Maltese | 42 | M | 3 | 404 | H | STI | Y | Odontogenic cyst | Y | T/I extraction and curettage | N | 12 | Cl and Rx | Healed |
| 3 | | Dog | Boxer | 24 | M | 33.5 | 405 | V | STI | Y | Odontogenic cyst | Y | T/I extraction and curettage | N | None | None | / |
| 4 | | Dog | German shepherd | 7 | M | 30 | 405 | H | STI | Y | Not performed | Y | Operculectomy | N | None | None | / |
| 5 | | Dog | Czechoslovakian wolf dog | 25 | M | 33 | 405 | MA | STI | Y | Odontogenic cyst | Y | T/I extraction and curettage | Dentin dysplasia | 2 | Cl and Rx | Healed |
| 6 | | Dog | Yorkshire terrier | 42 | M | 3.1 | 405 | MA | STI | N | Not performed | N | T/I extraction | N | None | None | / |
| 7 | | Dog | Labrador retriever | 11 | M | 35 | 405 | V | STI | N | Not performed | N | Operculectomy | N | None | None | / |
| 8 | 1 | Dog | Maltese | 7 | M | 4.2 | 204 | H | STI | N | Not performed | Y/N | T/I extraction | T/I 180° long assis | None | None | / |
| | 2 | Dog | Maltese | 7 | M | 4.2 | 305 | V | STI | N | Not performed | N | Operculectomy | N | None | None | / |
| 9 | | Dog | Boxer | 109 | F | 29.3 | 405 | DA | STI | Y | Not performed | Y | T/I extraction | N | 1 | Cl | Healed |
| 10 | | Dog | Boxer | 71 | M | 36.2 | 305 | V | STI | Y | Odontogenic cyst | Y | T/I extraction and curettage | N | 1 | Cl | Healed |
| 11 | | Dog | Boxer | 95 | FS | 25 | 305 | I | BI | Y | Odontogenic cyst | Y | T/I extraction and curettage | TR 305 | 1 | Cl | Healed |
| 12 | | Dog | Mixed breed | 122 | FS | 10.2 | 405 | MA | BI | N | Not performed | N | None | N | 3 | Cl | No change |
| 13 | 1 | Dog | Miniatture poodle | 7 | F | 5.8 | 305 | MA | STI | N | No cyst | N | Operculectomy | N | 3 | Cl and Rx | Healed |
| | 2 | Dog | Miniatture poodle | 7 | F | 5.8 | 405 | MA | STI | N | No cyst | N | Operculectomy | N | 3 | Cl and Rx | Healed |
| 14 | | Dog | Chihuahua | 49 | F | 2.6 | 404 | H | STI | Y | Odontogenic cyst | Y | T/I extraction and curettage | N | 1 | Cl | Healed |

| | | | | | | | | | | | | | | | | | |
|---|---|---|---|---|---|---|---|---|---|---|---|---|---|---|---|---|---|
| 15 | 1 | Dog | Pugs | 14 | M | 9.6 | 405 | V | STI | Y | Odontogenic cyst | Y | T/I extraction and curettage | N | 5 | Cl and Rx | Healed |
| | 2 | Dog | Pugs | 14 | M | 9.6 | 305 | H | BI | Y | Odontogenic cyst | Y | T/I extraction and curettage | N | 5 | Cl and Rx | Healed |
| | 3 | Dog | Pugs | 14 | M | 9.6 | 411 | H | BI | Y | Odontogenic cyst | Y | T/I extraction and curettage | N | 5 | Cl and Rx | Healed |
| 16 | | Dog | Mix breed | 93 | M | 11 | 305 | MA | STI | N | No cyst | N | T/I extraction | TR 305 | None | None | / |
| 17 | | Dog | Yorkshire Terrier | 83 | M | 3.3 | 411 | H | STI | N | Not performed | N | T/I extraction | N | 1 | Cl | Healed |
| 18 | | Dog | Bernese mountain dog | 49 | F | 33 | 310 | H | BI | N | Not performed | N | None | N | 0.5 | Cl | No change |
| 19 | 1 | Dog | Bernese mountain dog | 70 | FS | 42 | 208 | V | STI | N | Not performed | N | T/I extraction | Malformed 208 | None | None | |
| | 2 | Dog | Bernese mountain dog | 70 | FS | 42 | 410 | I | BI | N | Not performed | N | None | N | None | None | |
| 20 | | Dog | Boxer | 59 | F | 31.5 | 405 | V | BI | Y | Odontogenic cyst | Y | T/I extraction and curettage | N | 36 | Cl and Rx | Healed |
| 21 | 1 | Dog | Maltese | 19 | M | 5.4 | 305 | H | STI | Y | Odontogenic cyst | Y/N | T/I extraction | N | 1 | Cl | Healed |
| | 2 | Dog | Maltese | 19 | M | 5.4 | 311 | H | STI | N | Not performed | N | T/I extraction | N | 1 | Cl | Healed |
| | 3 | Dog | Maltese | 19 | M | 5.4 | 405 | H | STI | Y | Odontogenic cyst | Y | T/I extraction and curettage | N | 1 | Cl | Healed |
| | 4 | Dog | Maltese | 19 | M | 5.4 | 411 | H | STI | N | Not performed | N | T/I extraction | N | 1 | Cl | Healed |
| 22 | 1 | Dog | Labrador retriever | 9 | F | 22 | 401 | V | STI | N | Not performed | N | T/I extraction | N | 1 | Cl | Healed |
| | 2 | Dog | Labrador retriever | 9 | F | 22 | 301 SN | I | STI | N | Not performed | Y/N | T/I extraction | ABN 301 SN [illegible] | 1 | Cl | Healed |
| | 3 | Dog | Labrador retriever | 9 | F | 22 | 401 SN | I | STI | N | Not performed | Y/N | T/I extraction | ABN 401 SN (microdont) | 1 | Cl | Healed |
| 23 | | Dog | Mixed breed | 82 | MC | 25 | 405 | MA | STI | Y | Odontogenic cyst | Y/N | T/I extraction | N | 6 | Cl | Healed |
| 24 | | Dog | Mixed breed | 16 | M | 34.8 | 405 | V | STI | Y | Odontogenic cyst | Y | T/I extraction and curettage | N | None | None | / |
| 25 | | Dog | Italian shepherd | 24 | F | 35 | 405 SN | H | STI | Y | Not performed | Y | T/I extraction | N | 1 | Cl and Rx | Healed |
| 26 | | Dog | Boxer | 90 | FS | 35 | 305 | I | STI | Y | Odontogenic cyst | Y | T/I extraction and curettage | TR 305 | 12 | Cl and Rx | Healed |
| 27 | 1 | Dog | Labrador retriever | 19 | M | 35 | 305 | MA | STI | Y | Not performed | Y | T/I extraction | N | None | None | / |
| | 2 | Dog | Labrador retriever | 19 | M | 35 | 405 | MA | STI | N | Not performed | Y/N | T/I extraction | N | None | None | / |
| 28 | 1 | Dog | Labrador retriever | 35 | M | 35 | 305 | MA | BI | N | Not performed | N | None | TR 305 | 1 | Cl | No change |
| | 2 | Dog | Labrador retriever | 35 | M | 35 | 405 | MA | BI | N | Not performed | N | T/I extraction | TR 405 | 1 | Cl | Healed |
| 29 | 1 | Dog | Pekenese | 102 | M | 6.5 | 305 | H | STI | N | Not performed | Y/N | T/I extraction | N | 12 | Cl and Rx | Healed |
| | 2 | Dog | Pekenese | 102 | M | 6.5 | 405 | H | STI | N | Not performed | Y/N | T/I extraction | N | 12 | Cl and Rx | Healed |
| 30 | | Dog | Golden retriever | 15 | M | 29 | 305 | H | STI | N | Not performed | N | T/I extraction | N | 13 | Cl and Rx | Healed |

| | | | | | | | | | | | | | | | | | |
|---|---|---|---|---|---|---|---|---|---|---|---|---|---|---|---|---|---|
| 31 | | Dog | Bull mastiff | 12 | M | 59.5 | 305 | MA | STI | N | Not performed | Y/N | T/I extraction | N | None | None | / |
| 32 | 1 | Dog | Boxer | 40 | M | 30 | 305 | V | STI | Y | Odontogenic cyst | Y | T/I extraction and curettage | TR 305 | 15 | Cl and Rx | Healed |
| | 2 | Dog | Boxer | 40 | M | 30 | 405 | MA | STI | Y | Not performed | Y | T/I extraction and curettage | TR 405 | 13 | Cl and Rx | Healed |
| 33 | 1 | Dog | Miniature schnauzer | 54 | M | 8.6 | 305 | H | STI | N | Not performed | N | T/I extraction | N | 61 | Cl and Rx | Healed |
| | 2 | Dog | Miniature schnauzer | 54 | M | 8.6 | 405 | MA | STI | N | Not performed | N | T/I extraction | N | 61 | Cl and Rx | Healed |
| 34 | 1 | Dog | Boxer | 39 | M | 31 | 305 | H | STI | Y | Odontogenic cyst | Y | T/I extraction and curettage | TR 305 | 1 | Cl | Healed (second intention) |
| | 2 | Dog | Boxer | 39 | M | 31 | 405 | H | STI | Y | Odontogenic cyst | Y | T/I extraction and curettage | TR 405 | 1 | Cl | Healed (second intention) |
| 35 | 1 | Dog | Boxer | 110 | FS | 25 | 305 | V | BI | Y | OSA/cyst | Y | B/I | TR 305 | 3 | Cl and Rx | Clinical and Rx cyst/tumor enlargment |
| | 2 | Dog | Boxer | 110 | FS | 25 | 405 | V | BI | Y | Not performed | Y | None | TR 405 | 3 | Cl and Rx | Clinical and Rx cyst enlargment |
| 36 | 1 | Dog | Mixed breed | 114 | FS | 12.9 | 305 | MA | STI | N | Not performed | Y/N | T/I extraction and curettage | TR 305 | 24 | Cl | Healed |
| | 2 | Dog | Mixed breed | 114 | FS | 12.9 | 405 | MA | STI | N | Not performed | Y/N | T/I extraction and curettage | N | 24 | Cl | Healed |
| 37 | 1 | Dog | Chihuahua | 31 | M | 3 | 104 | H | STI | Y | Odontogenic cyst | Y | T/I extraction and curettage | N | 2 | Cl | Healed |
| | 2 | Dog | Chihuahua | 31 | M | 3 | 204 | H | STI | Y | Odontogenic cyst | Y | T/I extraction and curettage | N | 2 | Cl | Healed |
| | 3 | Dog | Chihuahua | 31 | M | 3 | 405 | H | STI | N | Not performed | N | None | N | 2 | Cl | No change |
| 38 | | Dog | Boxer | 79 | FS | 28 | 305 | MA | STI | N | Not performed | N | Operculectomy | N | 1 | Cl | Healed |
| 39 | | Dog | Boxer | 36 | M | 30.8 | 305 | H | STI | Y | Odontogenic cyst | Y | T/I extraction and curettage | TR 305 | 1 | Cl | Healed |
| 40 | | Dog | Bull terrier | 17 | M | 18 | 204 | H | BI | Y | Odontogenic cyst | N | T/I extraction and curettage | Malformed 204 | 19 | Cl and Rx | Healed |
| 41 | 1 | Dog | Chihuahua | 30 | FS | 1.8 | 305 | V | STI | N | Not performed | Y/N | T/I extraction | N | None | None | / |
| | 2 | Dog | Chihuahua | 30 | FS | 1.8 | 405 | V | BI | N | Not performed | Y/N | T/I extraction | N | None | None | / |
| 42 | | Dog | Pitbull | 34 | M | 32 | 405 | V | STI | N | Not performed | N | None | N | None | None | / |
| 43 | 1 | Dog | Irish setter | 116 | M | 38.9 | 305 | H | BI | N | Not performed | N | T/I extraction | N | None | None | / |
| | 2 | Dog | Irish setter | 116 | M | 38.9 | 405 | H | BI | N | Not performed | N | T/I extraction | N | None | None | / |
| 44 | | Dog | French bulldog | 16 | FS | 8.7 | 305 | MA | STI | N | Not performed | Y/N | None | N | 1 | Cl | No change |
| 45 | 1 | Dog | Boxer | 59 | M | 38 | 305 | MA | BI | N | Not performed | N | None | TR 305 | 1 | Cl | No change |
| | 2 | Dog | Boxer | 59 | M | 38 | 405 | MA | STI | Y | Odontodenic cyst | Y | T/I extraction and courettage | TR 405 | 1 | Cl | Healed |
| 46 | | Dog | English setter | 5 | M | 16 | 104 | H | STI | Y | CAA /cyst | Y | Rostral maxillectomy | N | 1 | Cl | Healed |

| | | | | | | | | | | | | | | | | | |
|---|---|---|---|---|---|---|---|---|---|---|---|---|---|---|---|---|---|
| 47 | | Dog | Pugs | 86 | M | 11.6 | 402 | H | BI | N | Not performed | N | T/I extraction | N | 27 | Cl and Rx | Healed |
| 48 | | Dog | Pugs | 59 | F | 10 | 405 | H | STI | Y | Odontogenic cyst | Y | T/I extraction and curettage | N | 27 | Cl and Rx | Healed |
| 49 | 1 | Dog | Boxer | 44 | FS | 34 | 405 | V | BI | Y | Odontogenic cyst | Y | T/I extraction and curettage | TR 405 | 68 | Cl and Rx | healed |
| | 2 | Dog | Boxer | 44 | FS | 34 | 205 SN | H | STI | N | Not performed | Y/N | T/I extraction | N | 68 | Cl and Rx | Healed |
| | 3 | Dog | Boxer | 44 | FS | 34 | 305 | V | STI | Y | Odontogenic cyst | Y | T/I extraction and curettage | TR 305 | 68 | Cl and Rx | Healed |
| 50 | 1 | Dog | Boxer | 18 | M | 34 | 305 | V | STI | Y | Not performed | Y | None | TR 305 | None | None | / |
| | 2 | Dog | Boxer | 18 | M | 34 | 405 | V | BI | Y | Not performed | Y | None | TR 405 | None | None | / |
| 51 | 1 | Dog | Shi-tzu | 86 | F | 5.9 | 311 | V | STI | N | Not performed | N | T/I extraction | N | None | None | / |
| | 2 | Dog | Shi-tzu | 86 | F | 5.9 | 411 | V | STI | N | Not performed | N | T/I extraction | N | None | None | / |
| 52 | | Dog | Maltese | 65 | F | 4.4 | 302 | H | STI | N | Not performed | N | T/I extraction | N | None | None | / |
| 53 | | Dog | Spitz | 72 | M | 4 | 305 | H | BI | N | Not performed | N | None | | 1 | Cl | No change |
| 54 | 1 | Dog | Boxer | 55 | M | 46 | 305 | I | BI | Y | Odontogenic cyst | Y | T/I extraction and courettage | TR 305 | 1 | Cl | Healed |
| | 2 | Dog | Boxer | 55 | M | 46 | 405 | I | STI | N | No cyst | N | None | TR 405 | 1 | Cl | No change |
| 55 | | Dog | Boxer | 43 | FS | 28 | 305 | H | STI | N | Not performed | Y/N | T/I extraction | Y TR 305 | | None | |
| 56 | | Dog | Pinscher | 37 | M | 4 | 405 | MA | STI | Y | Odontogenic cyst | N | Operculectomy | N | 1 | Cl | Healed |
| 57 | | Dog | Epagneul papillon | 17 | F | 2.7 | 411 | V | STI | N | Not performed | Y/N | T/I extraction | N | None | None | / |
| 58 | 1 | Dog | Chihuahua | 32 | M | 2 | 305 | H | STI | N | Not performed | N | T/I extraction | N | None | None | / |
| | 2 | Dog | Chihuahua | 32 | M | 2 | 405 | H | STI | N | Not performed | N | T/I extraction | N | None | None | / |
| 59 | 1 | Dog | Boxer | 104 | M | 40 | 305 | V | STI | Y | Not performed | Y | T/I extraction and curettage | TR 305 | 2 | Cl and Rx | Healed |
| | 2 | Dog | Boxer | 104 | M | 40 | 405 | V | STI | N | Not performed | Y/N | T/I extraction | TR 405 | 2 | Cl and Rx | Healed |
| 60 | 1 | Dog | Shi-tzu | 20 | FS | 6.2 | 305 | MA | STI | Y | Odontogenic cyst | Y | T/I extraction and curettage | N | 2 | Cl and Rx | Healed |
| | 2 | Dog | Shi-tzu | 20 | FS | 6.2 | 405 | MA | STI | Y | Odontogenic Cyst | Y | T/I extraction and curettage | N | 2 | Cl and Rx | Healed |
| 61 | | Dog | Boxer | 61 | M | 31.5 | 305 | H | STI | Y | Not performed | Y | None | N | 6 | Cl and Rx | Cyst enlargment and TR306 |
| 62 | 1 | Dog | Boxer | 40 | MC | 34 | 305 | H | STI | Y | Odontogenic Cyst | Y | T/I extraction and curettage | TR 305 | 2 | Cl and Rx | Healed |
| | 2 | Dog | Boxer | 40 | MC | 34 | 405 | MA | STI | Y | Odontogenic Cyst | Y | T/I extraction and curettage | N | 2 | Cl and Rx | Healed |
| | 3 | Dog | Boxer | 40 | MC | 34 | 105 SN | H | STI | Y | Odontogenic Cyst | Y | T/I extraction and curettage | N | 2 | Cl and Rx | Healed |
| | 4 | Dog | Boxer | 40 | MC | 34 | 205 SN | H | STI | Y | Odontogenic Cyst | Y | T/I extraction and curettage | N | 2 | Cl and Rx | Healed |

| | | | | | | | | | | | | | | | | |
|---|---|---|---|---|---|---|---|---|---|---|---|---|---|---|---|---|
| 63 | 1 | Dog | Chihuahua | 18 | F | 2.1 | 305 | MA | STI | N | No Cyst | N | T/I extraction | N | 1 | Cl | Healed (on phone) |
| | 2 | Dog | Chihuahua | 18 | F | 2.1 | 405 | MA | STI | N | No Cyst | N | T/I extraction | N | 0,5 | Cl | Healed (on phone) |
| 64 | | Dog | Labrador retriever | 34 | MC | 37 | 305 | MA | STI | Y | Odontogenic Cyst | Y | T/I extraction and curettage | TR 305 | 1 | Cl | Healed |
| 65 | | Dog | Miniature schnauzer | 112 | FS | 9.5 | 305 | MA | STI | N | Not performed | N | None | N | 3 | Cl | No change |
| 66 | | Dog | Labrador retriever | 127 | FS | 26.1 | 405 | V | STI | N | Not performed | N | T/I extraction | TR 405 | 26 | Cl | Healed |
| 67 | 1 | Dog | Spitz | 111 | M | 3 | 305 | MA | BI | N | Not performed | N | T/I extraction | TR 305 | 1 | Cl | Healed |
| | 2 | Dog | Spitz | 110 | M | 3 | 405 | V | STI | N | Not performed | N | T/I extraction | TR 405 | 1 | Cl | Healed |
| 68 | | Dog | Boxer | 92 | FS | 30 | 305 | I | BI | Y | Odontogenic cyst | Y | T/I extraction | TR 305 | 1 | Cl | Healed |
| 69 | | Dog | Maltese | 70 | F | 3.1 | 305 | MA | STI | N | Not performed | Y/N | T/I extraction | N | 1 | Cl | Healed |
